# Image memorability depends on interference in memory

**DOI:** 10.1038/s41598-025-21937-z

**Published:** 2025-10-21

**Authors:** Fernanda Morales-Calva, Aditi Velgekar, Michelle Sekili, Stephanie L. Leal

**Affiliations:** 1 Department of Psychological Sciences, Rice University, 6500 Main St., 77030 Houston, TX USA; 2 Department of Integrative Biology & Physiology, UCLA, 621 Charles E Young Dr S, Los Angeles, CA 90095 USA

**Keywords:** Neuroscience, Psychology, Psychology

## Abstract

Some experiences are better remembered than others by most people. This inherent characteristic of an event to be remembered or forgotten is known as memorability. However, it is unclear what drives certain experiences to be more memorable than others, and whether all aspects of an experience are better remembered. Interference across experiences is a major barrier that our memory system must overcome, yet we do not know how interference may impact the memorability of our experiences. Exploring this facet of memory may allow us to parse what drives certain experiences to be memorable or forgettable. To this end, we developed a memory task that varied image memorability and image interference during a memory test and measured target recognition - memory for repeated images, and lure discrimination - discernment of similar “lure” images, increasing interference in memory. While effects of memorability were evident for target recognition, the impact of memorability on lure discrimination depended on interference, where images with higher levels of interference did not benefit from memorability compared to lower levels of interference. Furthermore, these effects were time-dependent: memorability impacted memory immediately, but not after a longer time delay. These findings suggest that the concept of memorability depends on how and when memory is measured.

**Supplementary Information:**

The online version contains supplementary material available at 10.1038/s41598-025-21937-z.

## Introduction

Our everyday lives are full of rich and complex experiences. Some of these experiences tend to “stick” in our minds, while others are lost and forgotten. It seems intuitive that certain experiences are better remembered than others, such as highly emotional or salient events; however, there are experiences that are better remembered regardless of their saliency or emotional content. Memory research has primarily focused on participant performance on average; however, which properties of an experience are associated with improved memory may provide important information about how our memory system functions. Stimulus properties have been shown to play a role in determining whether or not it will be remembered, and these features may be even more consequential than other factors like individual differences^1^. The systematic variation with which some events are better remembered than others across individuals is known as *memorability*^2^.

Although not unique to scenes^3,4^, memorability of images has been the most frequently investigated^5^. Image memorability scores can be quantified using behavioral visual recognition memory tasks, where repeated and novel images are shown to participants who are asked to indicate ‘new’ versus ‘old’ images^6^. A memorability score for each image is then computed as the proportion of participants who correctly recognized a repeated image (0–1, where 1 indicates all participants remembered the image and 0 indicates no participants remember the image). These scores are consistent across samples and even in primates^7^, suggesting that memorability is largely independent of previous experience and consistent across individuals^8^. Further, memory performance declines with increased retention intervals, which could also be expected of behavioral memorability scores, which have been stated to decline log-linearly^9^. Memorability at specific intervals (e.g., ~ 15 images back, ~ 100 back) correlates with memorability at longer ones (~ 1000 back)^10,11^, suggesting that memorability is stable shortly after encoding and could be resistant to forgetting over time. However, most of these studies have analyzed differences in memory between ~ 5 to 40 min, with only a few looking at extended time periods for retention intervals^12^, which is more in line with real-world scenarios of remembering and forgetting^13^.

Recent advances in the training of deep neural networks and the acquisition of large human behavioral datasets have allowed for better characterization and understanding of memorability. For example, images with people in them are highly memorable in contrast to scenes of nature, which tend to have low memorability scores^14^. Brighter, colorful, larger, centered, and uncluttered images also tend to be more memorable^14^. However, there are still inconsistent findings regarding what drives memorability. For example, some studies have found that images with atypical content are more memorable^15^, while others have shown more typical images to be more memorable^1,16,17^. Another debate regarding memorability surrounds the idea that items with high emotional arousal are more memorable^9,18^, while other studies have no link between memorability and emotion^19,20^. Additionally, humans are fairly bad at determining what images will be more or less memorable; moreover, image memorability has been shown to be multifaceted, and much of memorability variance has remained unexplained in literature^21^. To understand the underlying neural mechanisms of memorability, neuroimaging studies have been conducted, which report that memorability can be classified by increases in neural activity, where ventral visual regions and medial temporal lobe regions, including perirhinal and parahippocampal cortices, are particularly sensitive to the memorability of a stimulus^17^, and neural activity tends to be more similar for memorable images, but more dissimilar for forgettable images^7,16^.

It has been proposed that hippocampal pattern separation, a neural computation that orthogonalizes overlapping experiences as distinct from one another, could be a candidate mechanism underlying memorability^1,10,21–23^. For example, it is possible that typicality would facilitate hippocampal pattern completion, or generalizing across similar experiences; nevertheless, because this is an area of active debate in the literature, studying memorability through a pattern separation framework could provide further evidence to better understand these effects. Similarly, neuroimaging studies showing more similar neural activity for memorable images, but more dissimilar activity for forgettable images is akin to work showing higher representational similarity during hippocampal pattern completion (generalization in the face of interference), and greater distinctiveness during hippocampal pattern separation (discrimination in the face of interference)^24^. To tax hippocampal pattern separation in humans, mnemonic discrimination tasks (MDTs) have been developed, which have been shown to activate hippocampal circuity and regions related to pattern separation^25,26^. MDTs allow for the measurement of both traditional recognition of repeated items (commonly used in the memorability literature) and lure discrimination, which measures a person’s ability to discriminate between similar (lure) stimuli with overlapping features^27^, increasing interference in memory^25^. We recently published a post-hoc analysis of memorability on mnemonic discrimination from an existing dataset using an emotional MDT, where we found interactions between memorability and image similarity that depended on time of testing^28^. However, this task was not designed with memorability in mind, thus, images were not balanced across memorability and included emotional images, which further impacts the dynamics between memorability and interference. To our knowledge, there is not an existing paradigm that has utilized a stimulus set that manipulates both image memorability and image similarity to examine their interactions.

Here, we designed a stimulus set and task design to merge the disparate fields of memorability and pattern separation by manipulating memorability by including memorable and forgettable images (as determined by target recognition measures) and stratified images by lure similarity (e.g., the presence of perceptual interference), an approach that has not been previously undertaken. Our daily experiences often involve similar activities, leading to overlap and interference across our memories. To enhance the authenticity of memory assessments, it is beneficial to create laboratory tasks that mirror natural retrieval processes, and which capture the intricacies of everyday experiences. Thus, the paradigm we have employed here allows us to mimic the interference we experience in our daily lives relative to traditional memory tasks that do not account for this important barrier our memory systems must face. By examining memory through a mnemonic discrimination paradigm, we propose a mechanistic account for the manifestation of these memorability trade-offs, potentially based on hippocampal pattern separation. As noted by previous studies^15^, extrinsic and intrinsic factors can influence how memorability manifests in specific contexts, supporting the idea that memorability is not a constant attribute but is influenced by various factors.

More broadly, this research contributes to our understanding of episodic memory, emphasizing the complex interplay between what we remember and forget. The definition of “memorability” is grounded in a general memory mechanism, such as target recognition. Nevertheless, human memory is more nuanced, such that not all information within an experience may be equally memorable. Certain aspects of an experience may be more memorable, while others may be more easily forgotten, all within the same context. By examining memorability in the context of hippocampal pattern separation, this allows us to examine more directly what type of information may be memorable or forgettable and holds significant implications for how we conceptualize what makes an experience memorable.

In the present study, we aimed to examine the interaction between image memorability and interference in a mnemonic discrimination paradigm using a stimulus set compiled with both memorability and pattern separation in mind. First, we curated a memorability-based image set using existing images quantified for memorability to be used in a mnemonic discrimination task. The inclusion of lure stimuli in mnemonic discrimination tasks requires hippocampal pattern separation to accurately discriminate them as unique, rather than repetitions, of encoded items; where the more similar a lure item is to its pair shown during encoding, the harder it will become to accurately discriminate during retrieval. MDTs tend to better introduce and measure the interference component of our mnemonic experiences, which make them more sensitive to cognitive dysfunction compared to traditional memory tasks used in memorability paradigms^26,29^. The MDT we created for the current study included varying levels of similarity (interference) as well as varying levels of memorability (see Supplementary Information 1 for full description of task development and stimuli selection). With this task, we examined how image memorability, lure similarity, and time of testing, impact both traditionally used target recognition measures, as well as lure discrimination measures, which have yet to be investigated in the context of memorability.

Given prior work, there are two potential accounts we are testing. First, memorable experiences may be more resistant to interference compared to forgettable experiences. In other words, if an experience is more memorable, it would be more resistant to disruption. Thus, for this account, we would predict that memorable images would be better remembered and discriminated compared to forgettable images on both target recognition and lure discrimination measures, respectively. Furthermore, the interaction between lower image similarity and higher memorability would lead to improved lure discrimination. These effects would be maintained or exaggerated across time delays, with those tested immediately outperforming those tested 24 h later.

Alternatively, if a memorable experience leads to a stronger memory trace, this could, in turn, make the memory more susceptible to overgeneralization and, thus, more prone to inference. If this account is true, retrieval would be more difficult for memorable images where there is higher overlap across representations (e.g., lures), which could lead to no difference between memorable and forgettable lure discrimination or even potentially a reversal, where forgettable images may show better lure discrimination compared to memorable images. Thus, if memorability boosts recognition memory (overgeneralization), it may negatively impact mnemonic discrimination. Furthermore, the amount of interference (i.e. similarity level) could be expected to interact with memorability, where higher levels of similarity and memorability would show worsened lure discrimination performance. Additionally, these effects would be maintained or exaggerated across time delays.

While previous memorability findings would predict enhanced target recognition and lure discrimination for memorable images across time delays, other accounts would predict stronger memory traces due to higher memorability. Such findings would demonstrate the complexity of our memory system and the need for more naturalistic paradigms to test both encoding and retrieval efforts that approximate real-world memory. Our current paradigm permits the testing of both of these accounts.

## Materials and methods

### Curation of the memorability-based mnemonic discrimination task

**Fig. 1:**
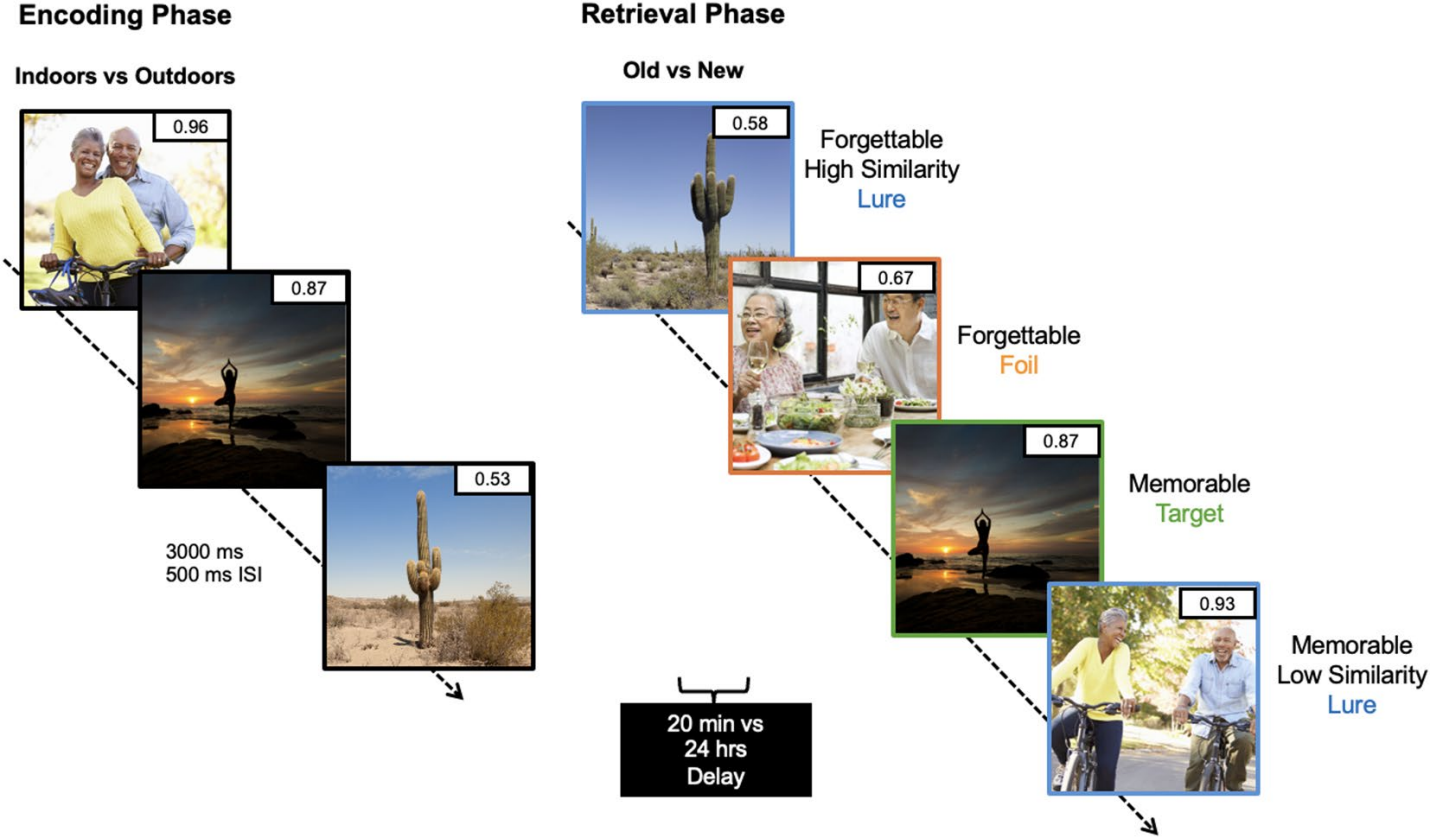
Memorability mnemonic discrimination task design. Participants were shown a series of images (3000 ms, 500 ms ISI) and were asked to identify them as “indoors” or “outdoors”. Participants were then given a surprise memory test either immediately or 24 h later. Images shown during retrieval were either the same (target), similar (lure), or new (foil). Permission was obtained for the use of all images in this figure, in which the images are licensed by Shutterstock, available at https://www.shutterstock.com/.

First, we curated a stimulus set to be used in a memorability-based mnemonic discrimination task using images from the *MemCat* dataset^14^, the second largest and the most up-to-date memorability image set (which includes with high-quality stimuli and memorability scores for every image)^2,9,30^ (see Supplementary Information 1–2 for complete details on task development). Briefly, *MemCat* was not designed to include perceptually and conceptually similar images, however, we manually searched the database to select 278 image pairs that were perceptually and conceptually similar to be rated on their similarity as potential lure stimulus pairs, a common feature of MDTs. Participants were instructed to rate the 278 image pairs on their similarity using a continuous scale ranging from 1 to 7, with boundary labels of 1 = unrelated, 3 = low similarity, 5 = high similarity, 7 = exact match. Participants could choose anywhere on the scale to determine their similarity rating and were not limited to whole numbers (e.g. interval measure, not ordinal). For example, in Fig. 1, the two images of cacti are perceptually and conceptually very similar but are different images. We also collected ratings of emotional arousal, valence, and familiarity, as these metrics have been associated with memorability in previous studies^10,18,31,32^, but were not the primary focus of the current study (see Supplementary Information 1 for detailed description on stimuli selection and categorization). Forty participants were recruited to provide the ratings on these stimuli. Informed consent was obtained from all participants, and the study procedures were approved by the Rice University Institutional Review Board (Study title: Cognitive, MRI, and PET studies of memory systems across the lifespan; Name of institution granting approval: Rice University; Protocol number: IRB-FY2020-6). All methods were performed in accordance with the relevant IRB guidelines and regulations and were performed in accordance with the Declaration of Helsinki. From the obtained ratings, we were able to select images in order to curate a rich, naturalistic, and diverse stimulus set to serve as stimuli in our memorability-based mnemonic discrimination task.

Based on previously validated mnemonic discrimination task designs^33–36^, we manually selected 80 image pairs that were perceptually and conceptually similar to be used as lures in the task (e.g., the cactus images in Fig. 1), across low (L), medium (M), and high (H) similarity levels, for a total of 160 images. Raw mean differences across similarity bins were significant and corresponded to large effect sizes [Cohen’s *d* = 2.62 (H vs. M), 3.94 (H vs. L), and 2.44 (M vs. L)]. Image pairs were generally balanced across the five main categories established by *MemCat* (animals, food, landscapes, sports, and vehicles) (Supplemental Tables S2 and S3). We also selected 80 images to be included as target images (repeated images during the memory test), which were unrelated in content to the lures (e.g., yoga images in Fig. 1), and 80 images as foils (novel images during the retrieval section of the memory test), also unrelated in content to either lures or targets (e.g., the couple having dinner image in Fig. 1 that was completely unrelated to any images shown during encoding). This yielded a total of 320 images to be included in the task, with 160 shown during the encoding period and 240 during retrieval (see Supplementary Information 1–2 for further detail). We designed the retrieval portion this way so that participant saw the exact same number of targets, foils, and lures during retrieval, thus better balancing and powering the behavioral results obtained from performance on these images.

#### Implementation of the memorability-based mnemonic discrimination task

After task development, 54 young adults (ages 18–35) were recruited from the Houston community through Rice University SONA, listservs, community pages, and flyers to undergo memory testing. Participants were compensated with course credit or gift cards for their participation in the study. This sample was independent from that involved in the stimuli ratings task to circumvent previous experience with the images. All participants were fluent in English and had normal to corrected vision. Two participants did not complete the study, leaving a final sample of 52 participants total. Testing took place from October 2022 to June 2023. Participant demographics can be found in Table 1.

**Table 1:** Participant demographics.

Variable	Immediate	Delay	Total
N	26	26	52
Age (M ± SD)	19.2 ± 1.1	21± 3.2	20.1 ± 2.5
Years of education (M ± SD)	12.6 ± 1.6	14.5± 2.8	13.5 ± 2.5
Gender M:F:NBi	11:14:1	12:14:0	23:28:1

Race & Ethnicity	Non-Latinx/o	Latinx/o
Arab/MENA	1	
Asian	21	
Asian & Pacific Islander	1	
Asian & White	2	1
Black	3	1
Black & White	1	1
White	14	5
White & Indigenous		1

Notation: M = Male, F = Female, NBi = Non-binary. MENA = Middle Eastern/North African.

During the encoding phase, participants were shown 160 images, in which half of the images were memorable and half were forgettable based on *MemCat* scores. Memorable versus forgettable categorization was determined using a threshold of 0.80 based on previous work^2,28,37^ and our dataset median (0.79). The images were on screen for 3000 ms with a 500 ms ISI. Participants were asked to determine whether the images took place “outdoors” or “indoors” to ensure they were paying attention to the stimuli. After encoding, participants were given a series of questionnaires related to a variety of lifestyle factors (see Supplementary Information 1). Either 20 min after encoding (*N* = 26, immediate) or 24 h later (*N* = 26, delay), participants were presented with a surprise memory test. During this retrieval phase, participants were shown a series of images (*N* = 240) which included 80 repeated images from the encoding phase (targets); 80 new, unrelated images (foils); and 80 similar, but not identical images (lures) to the other 80 images shown during encoding that were not targets (see Fig. 1). All participants were shown the same images during encoding and retrieval, but the order in which these were presented was randomized for each participant to avoid presentation bias.

Participants were instructed to categorize each image in the retrieval phase as “old” if the image was exactly the same as one shown during the encoding phase, or “new” if the image was new or different in any way from images shown during encoding. To measure memory at increasing interference levels, we split performance on lure images into three similarity levels - low, medium, and high similarity - using the similarity ratings obtained from the sample in the ratings task (see Table S4 for stimuli breakdown), where similarity categories were established based on participant ratings on how conceptually and visually similar image pairs were to each other (see Supplementary information 1 and 2 for additional information). After retrieval, all participants were asked to complete a series of neuropsychological tests to ensure normal cognition (see Supplementary Information 3).

### Memory measures of interest

The mnemonic discrimination task yields two key memory measures of interest that are calculated within individuals and then averaged across participants: *lure discrimination*, a behavioral putative correlate of hippocampal pattern separation^38,39^, and *target recognition*, a traditional memory measure for repeated images. Target recognition was measured by a discriminability index (*d’*), a signal detection measure, defined as the difference between the z-score normalization (*z*) of the proportion (*p*) of target (old) stimuli judged as ‘old’ (hit), and the z-score normalization (*z*) of the proportion of foils (*p*) (novel) stimuli judged as ‘old’ (false alarm), to correct for response bias, calculated as [*z(p(‘Old’|Target)) – z(p(‘Old’|Foil)]).* Z scores were calculated as z = (X – µ) / σ, where X was the individual score, µ was the mean of the score in the sample, and σ was the standard deviation of the measure in the sample. Lure Discrimination Index (LDI) was calculated as [*p(‘New’|Lure) – p(‘New’|Target)]*, which calculates the proportion of responding ‘new’ to a (similar) lure (correct rejection) and correcting for response bias by subtracting out responding ‘new’ to a target (old) stimulus (miss). This provides a behavioral index that taxes hippocampal pattern separation^29,40^ and has been used extensively in the field. While LDI is not a signal detection measure, one can calculate a Lure d’ as *z(p(‘Old’|Target)) – z(‘Old’|Lure)*. We replicate the reported LDI findings using Lure d’ (Supplementary Information 4), pointing towards the validity of LDI. Our memory measures of interest were calculated across levels of memorability (memorable, forgettable) derived at the image-level, and levels of lure similarity (low, medium, high) for LDI within participants.

### Statistical analyses

Statistical analyses were conducted using JASP 0.16.2^41^ statistical software. All data was examined for outliers where any observation more than three standard deviations from the sample mean was considered an outlier. No data was identified as an outlier. Student’s t-test was used to determine the difference between categories and groups, when Levene’s test of equality of variances was significant, Welch’s t-test was performed. Repeated-measures tests were corrected for non-sphericity violations as measured by Mauchly’s test using Greenhouse–Geisser correction. To determine the strength of the found relationships, Cohen’s d and partial eta squared (η_p_^2^) are reported for effect sizes. *Post-hoc* contrasts were conducted using Bonferroni correction or Scheffé’s method in SPSS^42^, where appropriate. Statistical values were considered significant at a final corrected alpha level of 0.05, which controlled for Type I error. Sample sizes were determined based on previous mnemonic discrimination paradigms^35,36,43,44^, but we also conducted *post-hoc* effect size sensitivity analyses using G*Power 3.1^45^ to explore the effect sizes we could detect with 80% power given our sample size (*N* = 52) and alpha of 0.05. For our analyses of variance and difference between means, we were powered to detect medium to large effects across groups (d = 0.79, f = 0.45)^46,47^. At the individual group level (*N* = 26), we were powered to detect large effects (f = − 0.59)^46,47^.

Given our lack of sensitivity to detect small and medium effects given our sample size, we also performed Bayesian statistics to explore whether any of our null results were more likely given the data without using a binary method such as null hypothesis significance testing (see Supplementary Information 5). All Bayesian analyses were conducted in JASP using the software’s default priors given no strong prior information was available to inform the analysis. Specifically, for t-tests and ANOVAs, JASP employs a Cauchy prior distribution centered at zero with a scale parameter of 0.707 for effect sizes, and a prior model probability of 0.5 for both the null and alternative hypotheses. Evidence was quantified using Bayes factors (BF₁₀), which represent the relative likelihood of the observed data under the alternative hypothesis compared to the null. All Bayes factors (BF₁₀) for the main analyses are summarized in Table S5, along with qualitative interpretations.

## Results

### Replication of target recognition and lure discrimination performance overall

**Fig. 2:**
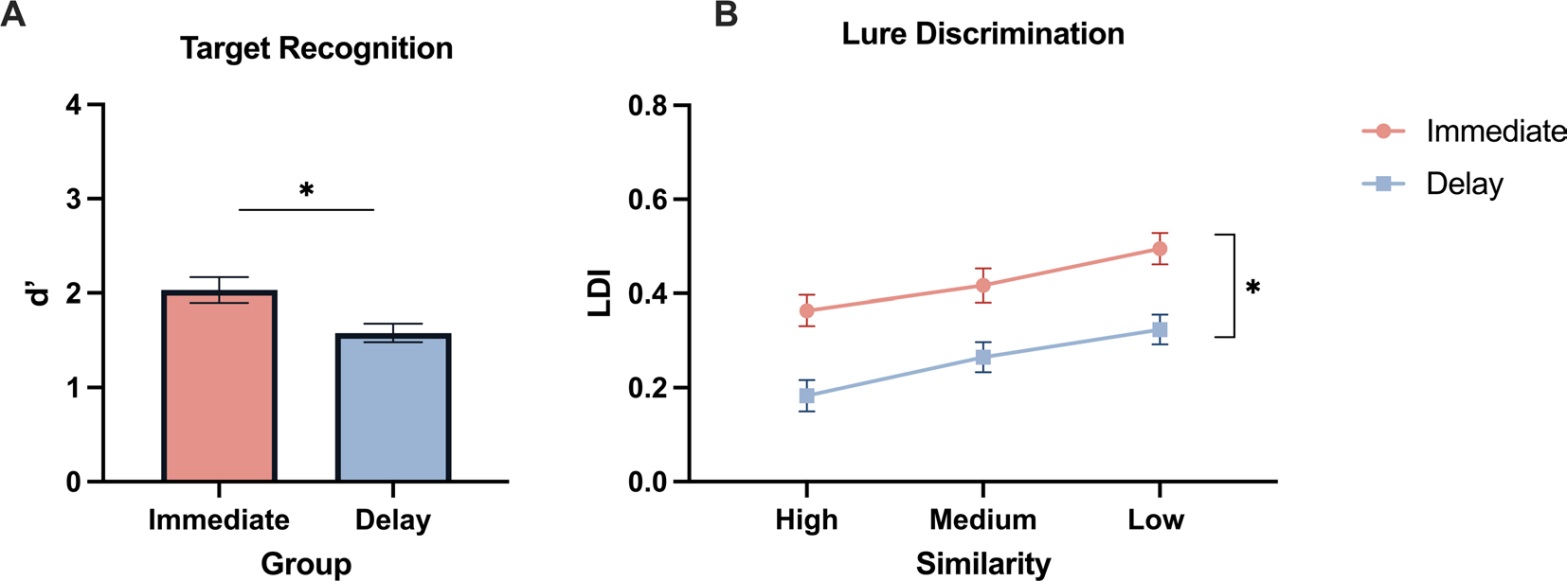
Target recognition and lure discrimination performance immediately and 24-hours later. A) Average target recognition (d’) in immediate (pink) and 24-hour delay (blue) groups. B) Average lure discrimination index (LDI) in immediate (pink) and 24-hour delay (blue) groups for low, medium, and high similarity images. Error bars represent SEM. * = *p* ≤ .05. Immediate *N* = 26, delay *N* = 26.

First, to ensure our novel mnemonic discrimination task replicated prior work, we examined differences in target recognition (*d’*) performance across immediate and delay groups using an independent samples t-test. As expected, target recognition was worse after a 24 h delay [*t*(*50)* = 2.72, *p* = .009, d = 0.29] (Fig. 2A). For lure discrimination, we performed a repeated-measures ANOVA with lure similarity (high, medium, low) as the within subjects’ factor and group (immediate, delay) as the between-subjects factor. As expected, there was a main effect of similarity [*F(2*,*100) =* 63.96, *p* < .001, η_p_^2^ = 0.56], with better lure discrimination as similarity levels decrease (e.g. less interference) [*F(1*,*50)* = 108.09, *p* < .001,η_p_^2^ = 0.68]. We found a main effect of group [*F(1*,*50)* = 14.032, *p* < .001, η_p_^2^ = 0.219], with better lure discrimination when tested immediately compared to a 24-hour delay (Fig. 2B). There was no interaction between similarity and group (*p* = .49). These findings confirm that our task design is tapping into established effects in mnemonic discrimination, as it replicated previous behavioral results^25^.

### Differential effects of memorability across memory measures

**Fig. 3:**
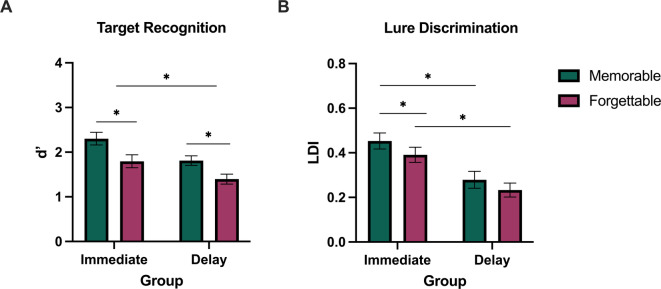
The effect of memorability on target recognition and lure discrimination immediately and 24 hours later. A) Average target recognition (d’) in immediate and 24-hour delay groups for memorable (teal) and forgettable (magenta) images. B) Average lure discrimination index (LDI) in immediate and delay groups for memorable (teal) and forgettable (magenta) images. Error bars represent SEM. * = *p* < .05. Immediate *N* = 26, delay *N* = 26.

To analyze the effects of memorability on target recognition, we performed a repeated measures ANOVA with memorability (memorable, forgettable) as the within subjects’ factor and group (immediate, delay) as the between-subjects factor. There was a significant effect of memorability, where memorable images were significantly better remembered than forgettable ones [*F(1*,*50) =* 37.30, *p* < .001, η_p_^2^ = .43]. We also found a main effect of group [*F(1*,*50) =* 7.31, *p* = .009, η_p_^2^ = .13], with those tested immediately outperforming those tested after 24 hours across both memorable [*t(50) =* 2.76, *p* = .008, d = .75] and forgettable [*t(50) =* 2.17, *p* = .035, d = .61] target recognition (Fig. 3A). There was no significant interaction between memorability and group (*p* = .54). Target recognition (*d’)* is how memorability has historically been measured, and these results align with previous memorability findings^13^.

To explore the effects of memorability on lure discrimination, we conducted a repeated measures ANOVA with memorability (memorable, forgettable) as the within subjects’ factor and group (immediate, delay) as the between-subjects factor for overall lure discrimination performance. We found a significant main effect of memorability, with memorable items being more easily discriminated than forgettable ones [*F(1*,*50) =* 7.75, *p* = .008, η_p_^2^ = 0.13]. We also found a main effect of group [*F(1*,*50) =* 13.32, *p* < .001, η_p_^2^ = 0.21], where those tested immediately outperformed those tested after 24-hours across both memorable [*t(50) =* 3.32, *p* = .002, d = 0.92] and forgettable [*t(50) =* 3.41, *p* = .001, d = 0.95] lure discrimination. There was no significant interaction between memorability and group (*p* = .67) (Fig. 3B). However, when examining the groups independently, we found that memorable images were better discriminated than forgettable ones in the immediate group [*t(25) =* 2.62, *p* = .015, d = 0.51], but not in the 24 h delay group [*t(25) =* 1.49, *p* = .149, d = 0.29], suggesting the main effect of memorability from the overall ANOVA was driven by those tested immediately and that the facilitating effect of discrimination for memorable items may be lost after extended time delays.

### Memorability and similarity interact to facilitate lure discrimination when tested immediately

**Fig. 4:**
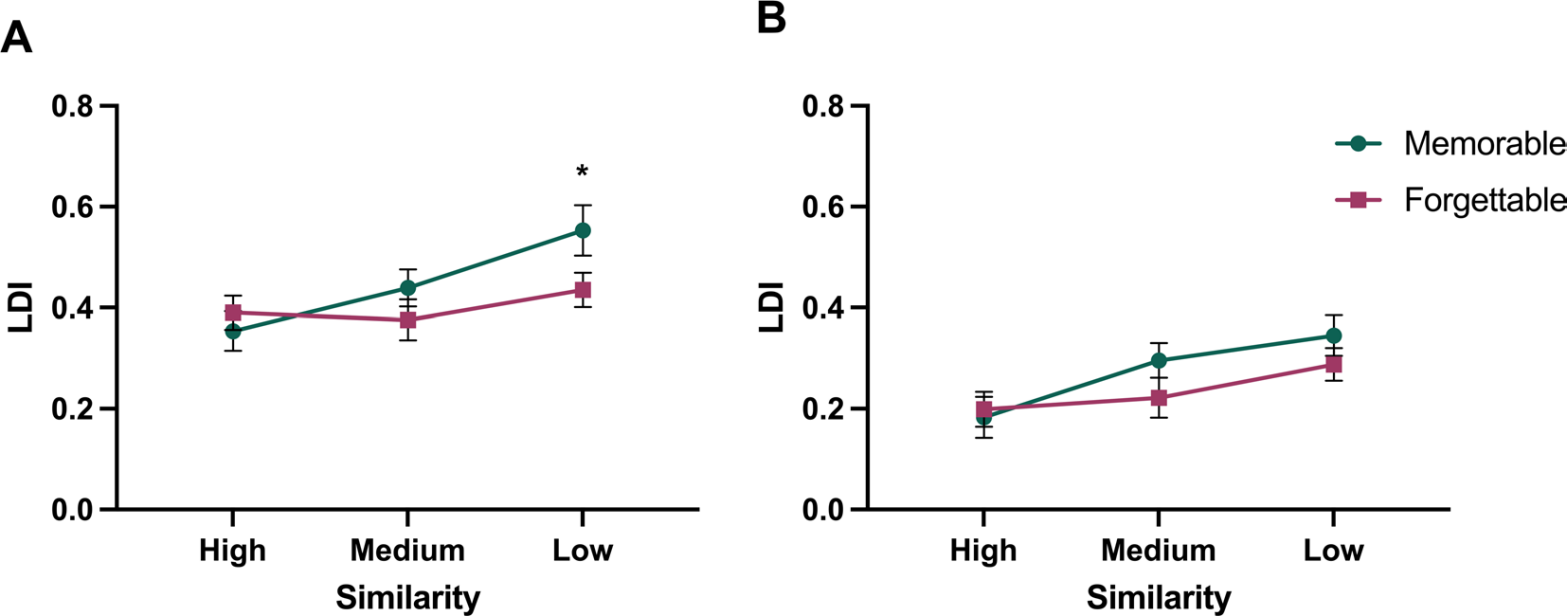
Interactions between memorability and lure similarity when tested immediately and 24 h later. Average lure discrimination index (LDI) in immediate (A) and delay (B) groups for memorable (teal) and forgettable (magenta) images. Error bars represent SEM. * *p* < .05. Immediate *N* = 26, delay *N* = 26.

To analyze the interaction between memorability and lure similarity, we conducted a repeated measures ANOVA with memorability (memorable, forgettable) and lure similarity (high, medium, low) as within-subjects factors within each group. In the immediate group, we found a significant main effect of similarity [*F(2*,*50) =* 15.95, *p* < .001, η_p_^2^ = 0.39]. Post-hoc Scheffé contrasts revealed a linear relationship, where high similarity images were the most difficult to discriminate, followed by medium, and then low similarity images [*F(1*,* 25)* = 34.24, *p* < .001, η_p_^2^ = 0.58]. Furthermore, there was a significant interaction between memorability and similarity [*F(2*,*50) =* 5.89, *p* = .012, η_p_^2^ = 0.19], such that as similarity decreased, lure discrimination increased, but only for memorable images relative to forgettable ones [*F(1*,*25)* = 21.62, *p* < .001, η_p_^2^ = 0.46] (Fig. 4A). We also found a marginal main effect of memorability in the expected direction [*F(1*,*25) =* 3.99, *p* = .057, η_p_^2^ = 0.14].

In the delay group, there was a significant main effect of similarity [*F(2*,*50) =* 35.52, *p* < .001, η_p_^2^ = 0.59], where the previously stated linear relationship (low > medium > high) was also present [*F*(1, 25) = 82.91, *p* < .001, η_p_^2^ = 0.77]. However, there was no significant main effect of memorability [*F(1*,*25) =* 2.23, *p* = .150, η_p_^2^ = 0.08] nor an interaction between memorability and similarity [*F(2*,*50) =* 2.25, *p* = .116, η_p_^2^ = 0.08] (Fig. 4B).

When conducting an ANOVA with group included as a between-subjects factor, this revealed the same main effects of memorability [*F(1*,*50) =* 6.65, *p* = .013, η_p_^2^ = 0.12], similarity [*F(2*,*100) =* 43.43, *p* < .001, η_p_^2^ = 0.46], and memorability and similarity [*F(2*,* 100) =* 7.91, *p* < .001, η_p_^2^ = 0.14], but additionally a significant main effect of group [*F(1*,*50) =* 13.59, *p* < .001, η_p_^2^ = 0.21], in which participants tested immediately showed better lure discrimination compared to those tested 24 h later. However, the interactions between memorability and group (*p* = .89), similarity and group (*p* = .49), and the three-way interaction between memorability, similarity, and group (*p* = .51) were not significant (all *p’*s > 0.05).

## Discussion

Using an existing memorability image set (*MemCat*) and an independent rater sample for image similarity, we designed a stimulus set to be used in a memorability-based mnemonic discrimination task. While previous work has shown that memorability can impact mnemonic discrimination^28^, these results were based on a post-hoc analysis of a task designed without controlling for memorability of the images. Here, we curated a stimulus set with both memorability and lure similarity in mind. The inclusion of lure images rated for similarity has been previously shown to tax hippocampal pattern separation. MDTs have been shown to be more sensitive to memory impairment compared to traditional recognition memory tasks^25^, the primary means by which memorability has been measured. Through the development of this stimulus set and task, we explored the interaction between image memorability and lure similarity and how they may play a role in making an experience memorable or forgettable. We found differential effects of memorability, where better memory for memorable images was observed for target recognition, as expected, while the effect of memorability on lure discrimination depended on lure similarity, with lower similarity images (less interference) showing the strongest effect of memorability. These results highlight that highly memorable experiences do not uniformly enhance memory performance and provides novel approach in which to consider memorability. This paradigm enables future studies to examine the neural mechanisms underlying these effects given the sensitivity of mnemonic discrimination tasks to hippocampal subfield activity^29,48^.

One account predicted that memorable images would be better remembered and discriminated compared to forgettable images on both target recognition and lure discrimination measures^49^. Indeed, we found that memorable images were more easily recognized (target recognition) and discriminated (lure discrimination) than forgettable ones. Overall, these findings provide support for a facilitating effect of memorability on mnemonic discrimination. However, when examining the interaction between memorability and similarity, memorable images were only better discriminated as similarity decreased (i.e. low similarity), and forgettable images showed no difference in lure discrimination across similarity levels. This finding is in line with the account that both lower similarity and higher memorability facilitate lure discrimination. Thus, memorability, in addition to lure similarity, are important predictors of memory performance. It is interesting that we do not see any evidence that lure similarity impacts discrimination of forgettable images, suggesting it is important to take memorability of the image, in addition to similarity, into account. This finding suggests that increased interference has little effect on further reducing the “forgettability” of an image, where lure discrimination measures can capture a more nuanced aspect of memorability that can inform our understanding of memory. Of note, the interaction between memorability and lure similarity was only evident when memory was tested immediately.

We also aimed to explore the effects of memorability with a longer time delay between encoding and retrieval, in which it is unclear whether memorability effects would be maintained after 24 h, as most forgetting occurs after 24 hours^50^. Thus, including a delayed testing timepoint is essential for understanding how memorability may translate to real-world memory and forgetting. For target recognition, memorable images were better remembered even after 24 h. This result is in line with previous studies looking at memorability after extended delays^11^, however, most of these studies have analyzed differences between ~ 5 to 40 min and not at extended time periods for retention intervals^13^. We did not observe lasting effects of memorability on lure discrimination after a 24-hour delay, suggesting the influences of memorability on lure discrimination may be lost after extended time intervals. Previous studies have shown that mnemonic discrimination is not modulated by the passage of time, rather by interference in memory^51^, pointing towards a role of memorability in modulating our findings. There may be a unique set of criteria that drives memorability for detailed information relative to general or gist information, or memorability for information after longer time delays. However, it is important to note that we did not find a three-way interaction between time delay, similarity, and memorability, where a larger sample may be needed to better examine these complex interacting effects. Thus, we cannot make any strong conclusions about whether the effect of memorability is lost after 24 h unless we are better powered to examine this interaction with larger sample sizes.

Examining the neural mechanisms underlying memorability could provide insights into the interactions between perception and memory. Given that our results are purely behavioral; neural data is required to make claims about the underlying neural mechanisms that may underlie our findings. It has been proposed that hippocampal pattern separation could be a candidate mechanism underlying memorability^52^. High-resolution neuroimaging during task performance will be important to measure to characterize the neurobiological mechanisms underlying our reported effects; namely, how hippocampal subfield activity and connectivity may play a role in the memorability of our experiences. We hypothesize there could be higher representational similarity in DG/CA3 during the encoding of memorable compared to forgettable images. We expect increased DG/CA3 subfield activity during conditions with increased interference for memorable compared to forgettable images, which is more sensitive to lure discrimination compared to other hippocampal subfields (e.g., CA1), and greater activity in ventral visual regions and the perirhinal and parahippocampal cortices during accurate target recognition of memorable compared to forgettable images. However, over time, such as after a 24-hour delay, we may see a shift from hippocampal to more neocortical activation that may or may not be impacted on memorability depending on the type of memory being measured (e.g., target recognition vs. lure discrimination). We would also expect higher connectivity between the medial temporal lobe and the ventral visual areas for memorable compared to forgettable images.

It is important to note some limitations of the current study. First, most of the stimuli used in the current study were relatively neutral in their content. Previous research has shown that images with emotional content tend to have higher memorability scores^9^. Recently, we analyzed an existing dataset that used an emotional MDT and divided stimuli into memorable and forgettable categories as determined by a convolutional neural network (CNN)^28^. We found that only lure discrimination showed an interaction between emotion and memorability, in which forgettable neutral images showed better lure discrimination compared to more memorable images, highlighting that careful consideration is required to determine what makes an image memorable and may depend on what aspects of the image are more memorable (e.g., gist vs. detail, emotional vs. neutral). We hypothesized that this effect was likely due to gist versus detail trade-offs often reported in the emotional modulation of memory literature^53^,such that emotional images may exhibit larger gist vs. detail trade-offs, where the central elements of emotional images (e.g., the gist) may be more memorable, while the peripheral, non-central elements (e.g., the details) of neutral, non-emotional images may be more memorable (or “forgettable” for the central/gist elements). However, in the current paradigm, we only include relatively neutral images, thus, the dynamics of how memorability and interference interact may be different.

Another limitation of the current task design is the thresholding of images into binary memorable and forgettable categories; however, we opted to use the 0.80 threshold in line with prior memorability work^2,15,37^ and as a way to increase power by including all images in our mnemonic discrimination task rather than excluding images near the threshold and only comparing extreme memorability conditions. We also used binning for lure similarity in line with previous mnemonic discrimination task work in terms of binning lure similarity^25,33,34,43^. Binning is necessary to perform the calculations needed to get d’ and LDI values in MDTs as we obtain performance at the participant level and not the stimulus level^25^. While performing calculations at the stimulus (instead of the participant) level is a possible way of obtaining continuous performance measures, these cannot be extrapolated into stimulus type, memorability, or similarity probabilities, hence an inability to obtain our memory measures of interest. Continuous performance allows for the representation of ‘Hit Rate’ per target image and ‘Correct Rejections’ per lure image. We examined continuous relationships between these measures at the stimulus level and report these correlations in Supplementary Information 6. Furthermore, our conceptualization of lure similarity was based on an independent sample’s ratings using a numerical scale, but more specificity in how similarity is measured and conceptualized by participants could better elucidate what drives some of the current results.

Furthermore, our immediate and delay groups were independent of each other (between-subjects design), so we could not directly compare within-subject performance immediately and after 24 h. This will be an important future direction to determine how memorability and memory performance in an individual may shift over 24 h. Another limitation already briefly discussed earlier is our relatively small sample size. While we did not conduct an a priori power analysis to detect effects of a specific size, our sample size is comparable to prior work using similar paradigms^33,54^, and the observed differences in similarity ratings between high, medium, and low similarity bins indicate that the effects across similarity bins are both statistically robust and practically meaningful. Given our relatively small sample size and corresponding limitations in power to detect small effects, it is particularly important to interpret reported effect sizes in relation to both theoretical predictions and practical significance. Our complementary use of Bayesian analyses, which can be advantageous for analyzing small sample sizes^55^, provided converging evidence when frequentist statistics alone might have overstated or understated the reliability of smaller effects. However, it is important to note that agreement between Bayes factors and frequentist p-values should not be interpreted as validation of the null or alternative hypotheses. Bayes factors are sensitive to the choice of prior and the available sample size, and phenomena such as the Jeffreys–Lindley paradox^55^ illustrate that Bayesian evidence can diverge sharply from p-value–based inference under certain conditions, and failure to pre-specify adequate sample sizes can limit the ability to detect effects of interest. Thus, our Bayesian analyses should be viewed as providing complementary evidence under specified priors, rather than as definitive confirmation of our frequentist results. Future studies should perform a priori power analyses to ensure small effects are detectable.

Two main characteristics of our sample might constrain the generality of our results. First, we chose to only recruit cognitively healthy young adults (ages 18–35) to establish baseline behavior as the current experimental paradigm was novel. However, future studies including a wider age range of participants and educational levels, will be important to determine whether these effects are maintained or altered with age. There has been evidence that participants with cognitive decline and mild cognitive impairment show consistent memorability for certain items, while other items show significant diagnostic abilities for these groups, where examining differences between which groups find certain images memorable can successfully categorize group membership with high success rate^56^. By identifying how memorability affects mnemonic discrimination, we may gain a better understanding of the memory deficits observed in aging and age-related cognitive decline, especially on mnemonic discrimination tasks^57^, as well as the neural underpinnings that may be impacted.

Moreover, we used memorability scores obtained from an existing dataset, where behavioral memorability scores were obtained from a European population^14^. Our sample was collected in the United States and had a more diverse racial and ethnic makeup (see Table 1). Although memorability has been reported to be consistent across observers^58^, most memorability studies have been performed in North American and European countries and populations, however more recent efforts have been made to explore memorability cross-culturally^59^. It is possible that some of the effects observed in the current sample are due to distinct cultural contexts, where memorability scores maybe inconsistent when stimuli such as food or landscapes are more infrequent with one group versus another. Previous studies have shown cross-cultural differences across the ability to discriminate lure images in mnemonic discrimination paradigms^60^. Although we tried to control for this variability by running all stimuli through a secondary validation for memorability scores (CNN), an important avenue moving forward will be validating memorability datasets, as well as our current findings, in a more culturally diverse groups to explore whether these results are generalizable.

Overall, our findings show that both similarity and memorability can be important predictors of memory performance; however, the impact of image memorability on memory is not uniform across memory measures, especially with increased interference. Studies examining memory, including studies of memorability, tend to use repeated and novel items during encoding and retrieval phases of memory testing. However, our everyday experiences are not as straightforward. The need to distinguish between similar experiences with overlapping content is an essential feature required for successful daily living. It is the inclusion of lure stimuli which makes our paradigm more naturalistic and novel when compared traditional memorability recognition tests. By testing the impact of image memorability on multiple types of memory measures through a mnemonic discrimination task, we aimed to better understand more specific (lure discrimination) versus general (target recognition) memory abilities related to memorability.

The findings presented here are important in highlighting the need to explore memorability beyond traditionally used recognition tasks, given that its effects may rely on differing memory mechanisms. In addition, a better understanding of memorability may lend itself to other domains, such as bettering educational outcomes for students, as well as a therapeutic or diagnostic tools for patients with disorders that may cause memory deficits. Moreover, intrinsic image properties such as memorability can influence remembering and forgetting. Through the investigation of the interaction between image memorability and lure similarity, we were able to examine the potential effects of these elements on traditional recognition and mnemonic discrimination.

## Supplementary Information

Below is the link to the electronic supplementary material.

Supplementary Material 1

## Data Availability

The data generated in the current study are available in a GitHub repository Morales-Calva, F., & L Leal, S. (2024). Memorability Based Mnemonic Discrimination Task (Version 1.0) [Computer software] https://github.com/lealmemorylab/memorability.
