# Supplementary material for: Image memorability depends on interference in memory
Source: Sci Rep. 2025 Oct 21;15:36597. doi: 10.1038/s41598-025-21937-z (PMC12540706; doi:10.1038/s41598-025-21937-z)
Supplement: Supplementary file 1 — Supplementary Material 1 [file 41598_2025_21937_MOESM1_ESM.docx]

**Supplement****ary Information**

**Supplementary Information 1: *Stimulus Set Development***

We sought to develop novel stimulus set to be used in a mnemonic discrimination paradigm. With this purpose, we used images from the *MemCat* dataset ^1^, the second largest and the most up-to-date memorability image set with high quality stimuli and memorability scores for every image. Memorability scores for each image in *MemCat* were obtained from ~99 participants in a behavioral visual repeat-detection memory task, where novel and repeated images were shown in a continuous stream and participants were instructed to indicate repeated images. A behavioral memorability score was computed as the proportion of participants (0 to 1, where 1 is most memorable and 0 is most forgettable) that correctly recognized a particular image upon it being repeated, *Memorability = Hit Rate - False Alarm Rate.*

*MemCat* is a category-based image set with 10,000 naturalistic images that represent five broad categories: food, animals, landscapes, sports, and vehicles. Inclusion of images across these categories allowed for variability in memorability scores (i.e., images of people are typically more memorable than images of landscapes) ^2–4^. Each category is further subdivided into multiple subcategories (i.e., bears, sandwiches, etc.). With the goal of choosing images with overlapping features to create lure pairs for the development of our mnemonic discrimination task, we manually selected two to three images from each of the 134 *MemCat* subcategories as possible items for our stimulus set where we aimed to obtain images with varying degrees of similarity. We also selected single images to serve as target and foil images in the memory task.

As an external validation for behavioral memorability scores reported for each *MemCat* image, we applied *ResMem* ^5^ on our selected stimuli, a convolutional neural network (CNN) that can analyze any image and predict a memorability score based on its edges, patterns, parts, and objects and was trained on a combination of behavioral memorability datasets ^6–8^. We compared the scores obtained from *ResMem* with the behavioral scores from *MemCat* and excluded any stimuli that showed a delta greater than 0.3 to include the most stable and reliable memorability scores for our selected images. After the exclusion of stimuli with high levels of variability in memorability scores, our stimulus set included 365 images.

From this image set, we collected similarity ratings of 278 image pairs, with images placed side by side. We included a range of what we considered to be high (N = 110) to low similarity (N = 122) image pairs as a starting point, as well as image pairs that had the same images side by side (identical pairs) (N = 23) and unrelated pairs (N = 23) to be included as attentional controls (see Supplemental Fig 1). Our goal was to design and balance an image set which varied both image memorability (memorable to forgettable), quantified through behavioral memorability scores reported in *MemCat*; and similarity of lure image pairs (high, to medium, to low similarity), quantified through participant ratings. For this we recruited participants to undergo a stimuli ratings task.

Participants (N = 40) underwent a computerized image ratings task for similarity of paired images, emotional valence, emotional arousal, subjective memorability, and familiarity of the images. Participants were recruited through Rice University SONA and received course credits for their participation. All participants were fluent in English and had normal to corrected vision. Informed consent was obtained from all participants, with all procedures approved by the Rice University Institutional Review Board. A breakdown of participant demographics can be observed in Table S1.

Because of the COVID-19 pandemic, testing was conducted online from February to April 2022 through the videoconference platform Zoom ^9^. All stimulus display and response collection routines were gathered using custom software written in PsychoPy ^10,11^. An Apple iMac was used to present the stimuli and record mouse responses via button press and remote sharing. Participants used the Zoom remote-control feature to control the mouse and keyboard of the recording computer. They were also instructed to set the task to full screen and hide their self and experimenter’s video panel to avoid distraction. The experimenter monitored task compliance.

Participants were instructed to rate the 278 image pairs on their similarity to each other using a continuous scale ranging from 1 to 7, with boundary labels of 1 = unrelated, 3 = low similarity, 5 = high similarity, 7 = exact match. Participants could choose anywhere on the scale to determine their similarity rating and were not limited to whole number choices (e.g. interval measure, not ordinal). All images were set to a width of 600 pixels and were presented horizontally aligned to each other in the center of the screen with a black background for 4000ms, during which participants needed to record their response, followed by an inter-stimulus interval (ISI) black background for 500ms. Image pairs were presented in randomized order across participants, and participants were given a break halfway through the task (~30 minutes total).

After rating image pairs for their similarity, participants were asked to rate 181-184 of the individual images on a continuous scale from 1 to 5 for emotional valence (1 = negative, 3 = neutral, 5 = positive), emotional arousal (1 = not at all, 3 = moderately, 5 = extremely), familiarity (1 = don't know what that is, 3 = moderately familiar, 5 = very familiar), and subjective memorability, framed as likelihood to remember a given image (1= very unlikely, 3 = somewhat, 5 = very likely). Images were presented one at a time on the center of the screen with a black background for 12000ms, time during which participants needed to record their response on the corresponding rating scale, each presented for 4000ms. Participants were given two breaks, each one after having rated 60 images in all four components. Images were presented in randomized order across participants. These ratings took ~40 minutes. To avoid participant fatigue, the individual ratings were divided into two groups, totaling 15 - 20 participant ratings per image across emotional arousal, emotional valence, familiarity, and subjective memorability.

**Supplementary Information 2: *Similarity ratings and stimulus selection***

Ratings for image pair similarity were obtained and averaged per pair across all participants. The obtained mean score was used to determine the similarity of each of the pairings on a continuous scale 1-7, where higher numbers reflect higher similarity across image pairs. Obtained values were equated to one of five possible categories based on the scale shown to participants: 1 – 1.9 = unrelated, 2 - 3.7 = low similarity, 3.8 – 4.2 = medium similarity, 4.3 - 5.9 = high similarity, and 6 - 7 = exact match. All the intended attentional controls were categorized as such; an indicator that participants responded to the task as required and expected. From the 232 rated image pairs, not including the attentional controls, 92 were categorized as high similarity (x̄ = 4.5 ± .28), 88 were categorized as medium similarity (x̄ = 3.9 ± .15), and 52 as low similarity (x̄ = 3.4 ± .23) by our sample based on the ranges noted above and based on the rating scale shown to participants (see Supplemental Figure 3A). We used these ratings to categorize images into similarity bins following prior paradigms that have created anywhere from 2-5 bins of similarity^12–14^.

Further, we obtained diverse ratings per each of the five major categories from *MemCat* (Supplemental Figure 3B). This allowed us to create a rich, naturalistic, and diverse stimulus set across all categories and subcategories to serve as lure stimuli in our memorability-based mnemonic discrimination task, where lure images were balanced across similarity (high, medium, and low). Ratings on familiarity, subjective memorability, emotional valence, and emotional arousal were obtained for all 365 individual images. We obtained a consistent spread of responses across all four categories, with most of our images being categorized as familiar (x̄ = 3.7), neutral (x̄ = 3.3), moderately arousing (x̄ = 2,4), and somewhat likely to be remembered (x̄ = 2.3) (Supplemental Figure 4). These ratings were not used for stimulus set selection, but we additionally performed correlational analyses between memorability scores and these additional variables. We found a significant Pearson’s *r* correlation between memorability score and arousal (*r* =0.116, *p* = .037, CI [0.007, 0.223]) and familiarity (*r*= 0.129, *p* =.021, CI [0.020, 0.236]). We found a marginal correlation between memorability scores and subjective memorability (r=0.109, *p* = .05, CI [-0.000, 0.213]) and no correlation between memorability and valence (r=0.038, p = .50, CI [-0.072, 0.147]) (see Supplemental Figure 1 for correlational plots).

All images were split into one of two memorability categories based on their *MemCat* memorability scores: memorable and forgettable. We split the stimuli into one of our two categories using a threshold of 0.80 based on previous work ^15–17^ and our dataset median (0.79). Given that no image ever scores 0, and few tend to score lower than 0.40, the threshold of 0.80 for distinguishing memorable and forgettable images allowed for the most power between memorability bins for our intended analyses, and higher number of stimuli to select from. Our final stimulus set included a range of memorability scores between 0.40 and .97. Additionally, we were able to select images from each of the five main *MemCat* subcategories so that the stimulus set was more diverse, naturalistic, and ecologically valid.

For the selection of lure pairs, we excluded any image pair in which the memorability score category was inconsistent across the stimuli comprising the pair (i.e., memorable baseline image paired with a forgettable lure image) to avoid confounding effects from this shift in category. Consequently, all selected image pairs maintained their assigned memorability category (memorable or forgettable) across our baseline-lure pairs. Moreover, we analyzed the mean difference in *MemCat* memorability scores for each baseline-lure pair [baseline *MemCat* memorability score subtracted from corresponding lure *MemCat* memorability score]. The difference score between baseline and lure pair memorability scores ranged from .001 to .201, with the mean difference score being .047. A paired t-test across baseline-lure pairs showed no significant differences across memorability scores [t(79) = 0.12, p=.90].

Next, we manually selected 80 pairs to be included in our task, with 26 pairs rated as high similarity (x̄ = 4.7 ± .33), 28 rated as medium similarity (x̄ = 4.0 ± .14), and 26 as low similarity (x̄ = 3.5 ± .21), by our previously described rater sample, for a total of 160 images, or 80 pairs. On average, high similarity pairs were rated 0.67 points higher than medium similarity pairs and 1.12 points higher than low similarity pairs, while medium pairs were 0.45 points higher than low pairs. These differences correspond to very large effect sizes (Cohen’s d = 2.62, 3.94, and 2.44, respectively) and represent shifts of up to ~16% of the full-scale range. Thus, the observed differences were not only statistically robust but also practically meaningful on the continuous similarity scale, supporting the validity of our approach. Image pairs were generally balanced across the five main categories established by *MemCat* (Supplemental Tables S2 and S3). We also selected 80 images to be included as target images (repeated images during the memory test) and 80 images as foils (novel images during the retrieval section of the memory test). This yielded a total of 320 images to be included in the task, with 160 shown during the encoding period and 240 during retrieval.

**Supplementary Information 3.1: Questionnaires**

Participants were administered a series of questionnaires related to lifestyle factors between the encoding and retrieval phases. They completed a 1) demographics form to collect information about race, ethnicity, age, sex, and years of education. All participants also completed: 2) the Stanford Sleepiness Scale (SSS) ^18^ to measure subjective alertness at the moment of evaluation, and 3) a modified version of the Pittsburgh Sleep Quality Index (PSQI) ^19^, which assesses sleep quality and disturbances over a one month interval. Participants in the 24-hour delay condition also completed 4) a second version of the modified PSQI asking about their sleep the previous night and the SSS again. We also collected the 5)Beck Depression Inventory (BDI) ^20^, and the 6) Beck Anxiety Inventory (BAI) ^21^ which ask about feelings of depression during the past two weeks and anxiety over the last month, respectively. Participants who indicated a positive response to the question related to suicidal ideation on the BDI were additionally administered a 7) Suicide Ideation Screening Questionnaire (SISQ) based on the Columbia Suicide Severity Rating Scale ^22^ and were given community resources after participation. Participants completed the 8) Attention Control Scale (ATTC) ^23^ to assess self-reported attention control, the 9) Adult ADHD Self-Report Scale (ASRS) ^24^, and 10) Adult ADHD Self-Report Screening Scale for DSM-5(ASRS-5) ^25^ which measure signs/symptoms of Attention-Deficit/Hyperactivity Disorder. They also completed an 11) informal learning style preference assessment adapted from ^26^, 12) a learning needs inventory to measure difficulties in learning settings, and the 13) Learning Styles Inventory developed by Canfield & Knight (1983) to measure instructional preferences. Finally, all participants filled out a 14) social media questionnaire developed in lab to measure social media use.

**Supplementary Information 3.2: *Neuropsychological tests***

Participants completed a battery of neuropsychological tests to assess general cognition and other cognitive functions. They completed: 1) the Digit Span Test which measures attention and working memory; 2) Trail Making Tests which measures attention, processing speed and task switching (Tombaugh, 2004); 3) the d2 test of attention ^28^ to measure selective and sustained attention; and 4) and the short form of Raven’s Progressive Matrices second edition, to measure high-level observational skills, reasoning and intellectual capacities. Three participants did not complete the Raven’s test due to time constraints during experimentation. Responses from a fourth participant were excluded given that their responses on the Raven’s test were identified as outliers given that they were more than three standard deviations from the mean.

**Supplemental Figures**

**Figure S1. Correlations between memorability scores and stimuli ratings.** We found a significant correlation between memorability score and memorability and familiarity. We found a marginal correlation between memorability scores and subjective memorability and no correlation between memorability and valence.


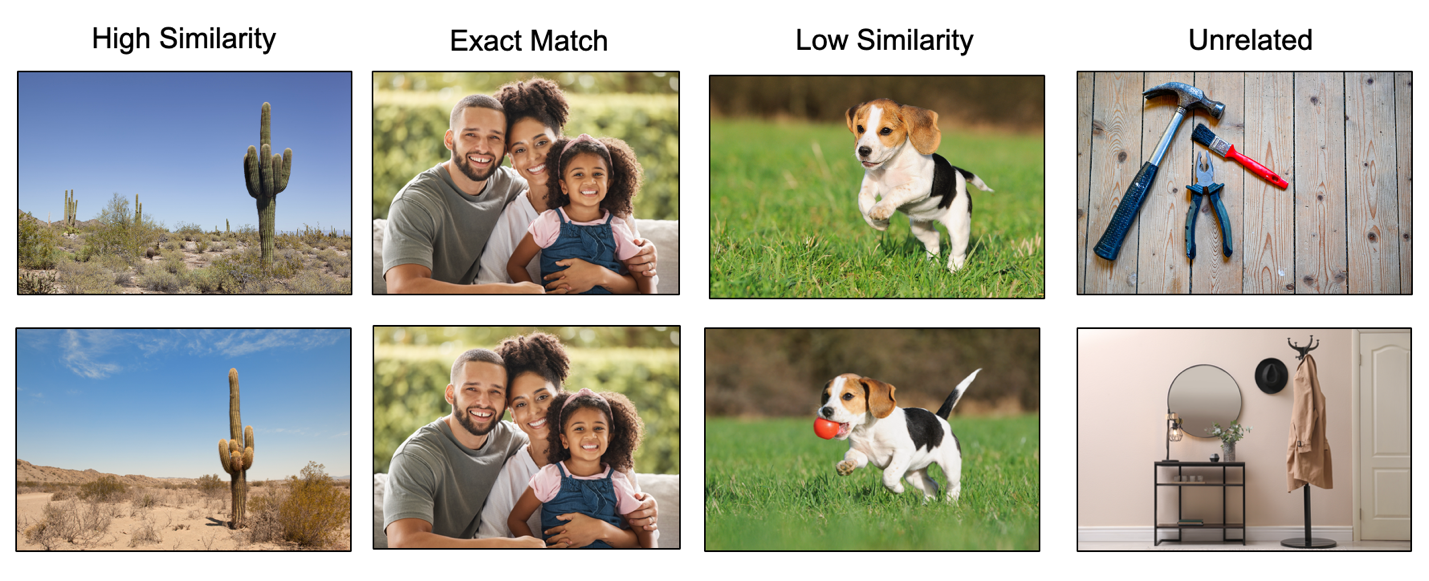


**Figure S2. Example pairings for stimuli ratings task.** During the task, participants were shown pre-selected image pairs ranging in similarity levels which included high, medium and low similarity pairs, repeated images (attentional control), and unrelated pairs (attentional control). Permission was obtained for the use of all images in this figure, in which the images are licensed by Shutterstock, available at https://www.shutterstock.com/.

**Figure S3. Similarity ratings.** A) Participants (N = 40) rated 278 pairs of images on how similar they thought they were to each other. 46 attentional controls (N = 23 unrelated, N = 23 exact match) were included. From the 232 rated image pairs, not including the attentional controls, 92 were categorized as high similarity (x̄ = 4.5 ± .28), 88 were categorized as medium similarity (x̄ = 3.9 ± .15), and 52 as low similarity (x̄ = 3.4 ± .23. X-axis represents similarity average obtained per image; Y-axis represents the category assigned based on the average similarity score. B) Participants rated 278 pairs of images on how similar they thought they were to each other. The figure displays the average ratings and categorization of the 232 image pairs that were not used as attentional controls, broken down by each of the five major MemCat categories. N = 40. X-axis represents similarity score average obtained per image; Y-axis represents the MemCat category the images belong to. Images categorized as low similarity are represented in orange, images categorized as medium similarity represented in blue, images categorized as high similarity presented in pink.


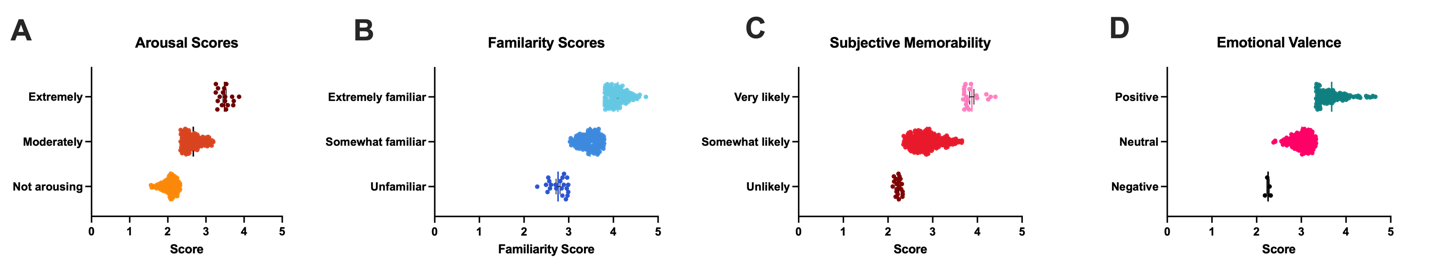
**Figure S4. Emotional arousal, familiarity, subjective memorability, and emotional valence scores obtained for stimuli from ratings task.** Ratings on A) emotional arousal, B) familiarity, C) subjective memorability, and D) emotional valence, were obtained for all 365 individual images. Participants rated half of the images on all four components. We obtained a consistent spread of responses for all four categories, with most of our images being categorized as A) moderately arousing (x̄ = 2,4), B) familiar (x̄ = 3.7), C) somewhat likely to be remembered (x̄ = 2.3) and D) neutral (x̄ = 3.3). The data collected from these categories was intended to give us further insights into our chosen stimulus set, however, these ratings were not used for stimulus set selection and thus not balanced for analysis of these variables.

**Supplementary Information 4: *Results using signal detection theory (d’) to capture lure discrimination in lieu of LDI***

We performed supplementary analyses to measure lure discrimination using lure d’, a SDT version of measuring lure discrimination We calculated lure d’ as Lure d’ = z(p(‘Old’|Target)) – z(‘Old’|Lure), in line with prior work^29^. This measure is often used when directly comparing target recognition and lure discrimination so that they are both d’ metrics, however, we are including the analysis of Lure d’ here to ensure that the results using LDI are replicated when measuring lure discrimination using a SDT framework.

***Replication of lure discrimination performance overall using lure d’***

First, to ensure our novel mnemonic discrimination task replicated prior work, we examined differences in lure d’ across immediate and delay groups, we performed a repeated-measures ANOVA with lure similarity (high, medium, low) as the within subjects’ factor and group (immediate, delay) as the between-subjects factor. As expected, there was a main effect of similarity [*F(2,100) =*58.884, *p* < .001, η_p_^2^ = .541], with better lure discrimination as similarity levels decrease (e.g. less interference). We found a main effect of group [*F(1,50)* = 13.047, *p* < .001, η_p_^2^ = .207], with better lure discrimination when tested immediately compared to a 24-hour delay. There was no interaction between similarity and group (*p* = .243). These findings replicate behavioral results using the LDI measure.

***Replication of the differential effects of memorability using lure d’***

To explore the effects of memorability on lure d’, we conducted a repeated measures ANOVA with memorability (memorable, forgettable) as the within subjects’ factor and group (immediate, delay) as the between-subjects factor for overall lure d’ performance. We found a significant main effect of memorability, with memorable items being more easily discriminated than forgettable ones [*F(1,50) =* 4.28, *p* = .04, η_p_^2^ = .079]. We also found a main effect of group [*F(1,50) =* 11.82, *p* = .001, η_p_^2^ = .19], where those tested immediately outperformed those tested after 24-hours across both memorable and forgettable lure d’. There was no significant interaction between memorability and group (*p* = .838). These findings replicate behavioral results using the LDI measure.

***Replication of the interaction between memorability and similarity to facilitate lure discrimination***

To analyze the interaction between memorability and lure similarity, we conducted a repeated measures ANOVA with memorability (memorable, forgettable) and lure similarity (high, medium, low) as within-subjects factors and group (immediate, delay) as a between-subjects factor. We found the same main effects of memorability [*F(1,50) =* 29.137, *p* < .001, η_p_^2^ = .368], similarity [*F(2,100) =* 8.23, *p* < .001, η_p_^2^ = .46], and memorability and similarity [*F(2, 100) =* 7.91, *p* < .001, η_p_^2^ = .54], but additionally a significant main effect of group [*F(1,50) =* 13.24, *p* <.001, η_p_^2^ = .21], in which participants tested immediately showed better lure d’ compared to those tested 24 hours later. However, the interactions between memorability and group (*p* = .87), similarity and group (*p* = .24), and the three-way interaction between memorability, similarity, and group (*p* = .99) were not significant (all *p’*s > .05). These findings replicate behavioral results using the LDI measure.

**Supplementary Information 5: Bayesian Inference**

**Methods**

In addition to our frequentist analyses, we also conducted Bayesian statistics using JASP (JASP Team, 2022) to supplement our frequentist results in order to explore how likely our models were relative to the null hypothesis. These analyses, which are frequentist adjacent, allow for an interpretation of the likelihood of the found effect being driven by the data as compared to null models. These analyses were motivated to further explore null results in our frequentist approach, given our lack of sensitivity to detect small effects. **We used the software’s default priors given no strong prior information was available to inform the analysis. Specifically, for t-tests and ANOVAs, JASP employs a Cauchy prior distribution centered at zero with a scale parameter of 0.707 for effect sizes, and a prior model probability of 0.5 for both the null and alternative hypotheses. Evidence was quantified using Bayes factors (BF₁₀), which represent the relative likelihood of the observed data under the alternative hypothesis compared to the null.** All Bayes factors (BF₁₀) for the main analyses are summarized in Table S5, along with qualitative interpretations. All error percentages were much less than the 20% rule of thumb (van Doorn et al., 2023), which indicates that the observed Bayes factors (BF) are stable. Moreover, we include the posterior inclusion probability through the inclusion BF, calculated as the change from prior to posterior inclusion odds matched across all candidate models, where all models with the interaction effect are compared to models with the same predictors except for the interaction effect as suggested by (Mathôt, 2017). This can be interpreted as the evidence in the data for including the predictor. We note that convergence between Bayes factors and frequentist statistics should not be interpreted as validation, as Bayes factors are sensitive to prior choice and sample size. In particular, the Jeffreys–Lindley paradox illustrates that apparent agreement can be misleading when sample size is not chosen *a priori* to ensure adequate sensitivity. Accordingly, the Bayes factors reported here are provided as complementary support under the specified priors rather than as confirmatory validation of p-values.

**Results**

***Target recognition and lure discrimination performance***

First, we examined differences in target recognition (*d’*) performance across immediate and delay groups using a Bayesian independent samples student t-test. We observed moderate evidence for differences in *d’* performance relative to the null model (BF_10_ = 5.24), where the immediate group performed better than the delay group. These results align with the frequentist results where we found a significant effect of time of testing, however the Bayesian t-test only provides moderate evidence for the effects of group.

For lure discrimination, we conducted a repeated-measures Bayesian ANOVA to examine the evidence provided by our model of lure similarity effects across groups, with similarity (low, medium, high) as the within-subjects factor and group (immediate, 24-hour delay) as the between-subjects factor. We observed extreme evidence (BF_10_ = 1.561x${10}^{17}$) that the model with two main effects (Similarity + Group) best predicted the data relative to the null model, the error term indicated the BF was stable. We then compared the analysis of effects through the BF inclusion, which was calculated as the change from prior inclusion odds to posterior inclusion odds for each component containing the effect to equivalent models stripped of the effect, or matched models. It revealed that Similarity (BF_inclusion_ = 2.73x${10}^{14}$) had an extreme effect on the differences in memory performance. We also observed that group (BF_inclusion_ = 31.38) allowed for a very strong conclusion about the effects of the differences in memory performance. The interaction, on the other hand, was not sufficiently informative to allow for a conclusion about the interaction (BF_inclusion_ = 0.71). These results align with the frequentist results where we found a significant effect of time of testing and no significant interaction. However, the Bayesian ANOVA only provides moderate evidence for the effects of memorability.

***Differential effects of memorability across memory measures***

To analyze the effects of memorability on target recognition, we performed a repeated measures Bayesian ANOVA with memorability (memorable, forgettable) as the within subjects’ factor and group (immediate, delay) as the between-subjects factor. We observed extreme evidence (BF_10_ = 729612.1) that the model with two main effects (Memorability + Group) best predicted the data relative to the null model, and the error term indicated the BF was stable. We then compared the analysis of effects through the BF inclusion across matched models. This analysis revealed that memorability (BF_inclusion_ = 93307.271) had an extreme effect in the differences in d’ performance. We observed that group (BF_inclusion_ = 7.11) allowed for moderate evidence for the differences in memory performance. The interaction was not sufficiently informative to allow for a conclusion about differences in memory performance (BF_inclusion_ = 0.659). When comparing these results with our frequentist analyses, we were able to find effects of memorability and group and a lack of evidence for a significant interaction.

To explore the effects of memorability on lure discrimination, we conducted a repeated measures Bayesian ANOVA with memorability (memorable, forgettable) as the within subjects’ factor and group (immediate, delay) as the between-subjects factor for overall lure discrimination performance. We observed extreme evidence (BF_10_ = 232.82) that the model with two main effects (Memorability + Group) best predicted the data relative to the null model, and the error term indicated the BF was stable. We then compared the analysis of effects through the BF inclusion and found that group (BF_inclusion_ = 36.58) had an extreme effect in the differences in LDI performance, while and memorability (BF_inclusion_ = 4.655) had moderate effects. The interaction between memorability*group (BF_inclusion_ = 0.97) did not display sufficient evidence for the differences in lure discrimination. When comparing these results with our frequentist analyses, we were able to find effects of group and memorability and a non-significant interaction. It is important to note that the found effects of memorability only had moderate support through the Bayesian approach.

***Memorability and similarity interact to facilitate lure discrimination when tested immediately***

To analyze the interaction between memorability and lure similarity, we conducted a repeated measures ANOVA with memorability (memorable, forgettable) and lure similarity (high, medium, low) as within-subjects factors within each group. In the immediate group, we observed extreme evidence (BF_10_ = 16713.202) that the model with the interaction and the main effects best predicted the data relative to the null model, and the error term indicated the BF was stable. We then compared the analysis of effects through the BF inclusion and found that similarity (BF_inclusion_ = 6075.79 had an extreme effect in the differences in LDI performance, memorability (BF_inclusion_ = 21.05) has very strong effects. Additionally, the interaction between memorability*similarity (BF_inclusion_ = 59.44) displayed very strong effects in the differences in lure discrimination. When comparing these results with our frequentist analyses, we were able to find effects of similarity, as well as a significant interaction, however, the evidence found for memorability in the Bayesian analyses was only marginal in the frequentists approach.

In the delay group, we found extreme evidence for the main effects plus the interaction (BF_10_ = 64630.15) best predicting the data relative to the null model. When comparing the analysis of effects through the BF inclusion factor, we found that similarity (BF_inclusion_ = 52406.88) had an extreme effect in the differences in immediate LDI performance. The main effect of memorability only showed anecdotal evidence for the effect (BF_inclusion_ = 1.62), while the interaction showed a moderate effect (BF_inclusion_ = 3.537). The findings align with our frequentists results, where we found an effect of similarity but no effect of memorability nor a significant interaction. Finally, we examined the effects of memorability and similarity in LDI across across testing times. We conducted a repeated-measures Bayesian ANOVA with memorability (memorable, forgettable) and similarity (low, medium, high) as within-subjects factors and group (immediate, delay) as the between-subjects factors. We observed extreme evidence (BF_10_ = 4.382 x${10}^{13}$) that the model with three main effects and the interaction between similarity and memorability (Memorability + Similarity + Group + similarity*memorability) best predicted the data relative to the null model, and the error term indicated the BF was stable. We then compared the analysis of effects through the BF inclusion and found that similarity (BF_inclusion_ = 1.014 x${10}^{11}$), memorability (BF_inclusion_ = 510), and their interaction (BF_inclusion_ = 634.687) provided extreme evidence for the differences in LDI performance. Additionally, group (BF_inclusion_ = 24.728) displayed strong evidence for the effects. All other interactions only displayed anecdotal evidence to allow for a conclusion about differences in memory performance (BF_inclusion_ < 1). These findings lend support to our frequentist results reported in the main body of the text, where we found effects of memorability, similarity and their interaction, as well as a main effect of group, with no other interactions showing significant trends.

**Supplementary Information 6: Examination of memorability, similarity, and memory continuously at the stimulus-level**

We also examined memorability continuously at the individual stimulus-level to determine whether memorability was associated with hit rate for target images and correct rejection rate for lure images. We found a significant correlation between memorability and target hit rate across both immediate and delay groups combined (r =.434, *p* <.001). Higher memorability scores were associated with higher target hit rate for repeated images, as expected based on memorability literature. When looking at the correlation within each group, we found significant correlations between target hit rate and memorability in the immediate (r =.4341 *p* <.001) and delay conditions (r =.34, *p* .002). When examining the relationship between memorability and lure correct rejection rate at the stimulus-level across both immediate and delay groups, we did not find a significant correlation between memorability and lure correct rejection rate (r = -0.183, *p* = .104), nor when looking at groups separately [Immediate (r =-.129 *p* = .252); delay (r =-.1993, *p* = .07)].

We also examined the relationship between similarity and lure correct rejection rate and found a significant negative correlation between similarity and lure correct rejection rate (r = -0.489, *p* <.001). Lower similarity scores were associated with higher lure correct rejections rates. This was true in the immediate (r = -0.468, *p* <.001) and delay (r = -0.431, *p* <.001) groups when looking at them separately.

**Supplemental Tables**

**Table S1. *Participant Demographics for Stimuli Ratings***

|  |  |  |
| --- | --- | --- |
| **N** | 40 |  |
| **Age** | 19.5 ± 1.3 |  |
| **Years of education** | 13.3 ± 1.4 |  |
| **Gender M : F** | 13 : 27 |  |
|  |  |  |
| **Race & Ethnicity** | Latino/x | Non-Latino/x |
| **Asian** |  | 14 |
| **Asian & Pacific Islander** |  | 1 |
| **Asian & White** |  | 1 |
| **Black** | 1 | 4 |
| **White** | 2 | 16 |

**Table S2. Stimuli selected for encoding phase of memorability-based mnemonic discrimination task**

| **Category** | **Memorable** | **Forgettable** | **Total** |
| --- | --- | --- | --- |
| **Baseline / Target** | **40** | **40** | **80** |
| animal | 8 | 8 | 16 |
| food | 13 | 6 | 19 |
| landscape | 4 | 9 | 13 |
| sports | 11 | 11 | 22 |
| vehicle | 4 | 6 | 10 |
| **Baseline Lure** | **41** | **39** | **80** |
| **High similarity** | **15** | **11** | **26** |
| animal | 5 | 1 | 6 |
| food | 3 | 0 | 3 |
| landscape | 1 | 5 | 6 |
| sports | 3 | 3 | 6 |
| vehicle | 3 | 2 | 5 |
| **Medium similarity** | **12** | **16** | **28** |
| animal | 1 | 1 | 2 |
| food | 4 | 1 | 5 |
| landscape | 1 | 4 | 5 |
| sports | 3 | 5 | 8 |
| vehicle | 3 | 5 | 8 |
| **Low similarity** | **14** | **12** | **26** |
| animal | 2 | 0 | 2 |
| food | 4 | 0 | 4 |
| landscape | 0 | 7 | 7 |
| sports | 4 | 3 | 7 |
| vehicle | 4 | 2 | 6 |
| **Grand Total** | **81** | **79** | **160** |

**Table S3. Stimuli selected for retrieval phase of memorability-based mnemonic discrimination task**

| **Category** | **Memorable** | **Forgettable** | **Total** |
| --- | --- | --- | --- |
| **Baseline/Target** | **40** | **40** | **80** |
| animal | 8 | 8 | 16 |
| food | 13 | 6 | 19 |
| landscape | 4 | 9 | 13 |
| sports | 11 | 11 | 22 |
| vehicle | 4 | 6 | 10 |
| **Lure** | **41** | **39** | **80** |
| **High similarity** | **15** | **11** | **26** |
| animal | 5 | 1 | 6 |
| food | 3 | 0 | 3 |
| landscape | 1 | 5 | 6 |
| sports | 3 | 3 | 6 |
| vehicle | 3 | 2 | 5 |
| **Medium similarity** | **12** | **16** | **28** |
| animal | 1 | 1 | 2 |
| food | 4 | 1 | 5 |
| landscape | 1 | 4 | 5 |
| sports | 3 | 5 | 8 |
| vehicle | 3 | 5 | 8 |
| **Low similarity** | **14** | **12** | **26** |
| animal | 2 | 0 | 2 |
| food | 4 | 0 | 4 |
| landscape | 0 | 7 | 7 |
| sports | 4 | 3 | 7 |
| vehicle | 4 | 2 | 6 |
| **Foil** | **39** | **41** | **80** |
| animal | 10 | 8 | 18 |
| food | 8 | 8 | 16 |
| landscape | 6 | 10 | 16 |
| sports | 8 | 8 | 16 |
| vehicle | 7 | 7 | 14 |
| **Grand Total** | **120** | **120** | **240** |

**Table S4. Results from additional questionnaires and neuropsychological testing.**

| Variable | Immediate |  | Delay |  |
| --- | --- | --- | --- | --- |
| Hyperactivity Symptoms (ASRS) | 21.85 ± | 4.97 | 19.57 ± | 4.41 |
| Inattention Symptoms (ASRS) | 27.62 ± | 6.36 | 27.23 ± | 5.32 |
| ADHD Symptoms (ASRS-5) | 8.04 ± | 3.09 | 8.19 | 3.41 |
| Attention Focusing (ATTC) | 22.85 ± | 4.72 | 22.50 ± | 3.85 |
| Attention Shifting (ATTC) | 26.65 ± | 5.16 | 27.11 ± | 3.59 |
| Attention Total (ATTC) | 49.50 ± | 8.34 | 49.62 ± | 5.90 |
| Anxiety (BAI) | 12.23 ± | 11.39 | 7.23 ± | 7.45 |
| Depression (BDI) | 12.08 ± | 7.26 | 8.88 ± | 8.25 |
| Concentration Performance (D2) | 241.19 ± | 41.07 | 228.19 ± | 30.49 |
| Errors of Omission (D2) | 13.77 ± | 13.57 | 19.08 ± | 11.74 |
| Errors of Commission (D2) | 0.77 ± | 1.63 | 0.69 ± | 1.83 |
| Numbers Processed (D2) | 577.62 ± | 71.65 | 565.92 ± | 42.24 |
| Total Errors (D2) | 563.08 ± | 71.58 | 546.15 ± | 48.10 |
| Digit Span Backward | 7.31 ± | 2.00 | 7.81 ± | 1.83 |
| Digit Span Forward | 11.19 ± | 2.35 | 11.46 ± | 1.79 |
| Learning Needs | 2.00 ± | 3.24 | 1.65 ± | 3.21 |
| Social Media Use* | 14.58 ± | 3.97 | 11.46 ± | 3.79 |
| Sleep Quality (PSQI) | 3.00 ± | 1.10 | 3.19 ± | 1.27 |

Mean and SD scores across questionnaires and neuropsychological tests across immediate and delay testing groups. Overall, we found no significant differences across groups (all *p’*s > .05) with the exception of social media use (**p* = .005).

**Table S5. Summarized Bayes Factor results and interpretations**

| **Effect / Comparison** | **BF₁₀** | **Interpretation** |
| --- | --- | --- |
| Target recognition (Immediate vs. Delay) | 5.24 | Moderate evidence for group effect |
| Lure discrimination – Similarity main effect | 2.73 × 10⁰ | Moderate evidence |
| Lure discrimination – Group main effect | 31.38 | Very strong evidence |
| Lure discrimination – Interaction (Similarity × Group) | 0.71 | Anecdotal evidence for null |
| Target recognition – Memorability main effect | 93,307.27 | Extreme evidence |
| Target recognition – Group main effect | 7.11 | Moderate evidence |
| Target recognition – Interaction (Memorability × Group) | 0.66 | Anecdotal evidence for null |
| Lure discrimination – Memorability main effect | 4.66 | Moderate evidence |
| Lure discrimination – Group main effect | 36.58 | Extreme evidence |
| Lure discrimination – Interaction (Memorability × Group) | 0.97 | Anecdotal evidence for null |
| Immediate group: Memorability × Similarity interaction | 59.44 | Very strong evidence |
| Delay group: Memorability main effect | 1.62 | Anecdotal evidence |
| Delay group: Memorability × Similarity interaction | 3.54 | Moderate evidence |
| Across groups: Memorability main effect | 510.0 | Extreme evidence |
| Across groups: Similarity main effect | 1.01 × 10³ | Extreme evidence |
| Across groups: Memorability × Similarity interaction | 634.69 | Extreme evidence |
| Across groups: Group main effect | 24.73 | Strong evidence |

**References**

1. Goetschalckx, L. & Wagemans, J. MemCat: a new category-based image set quantified on memorability. *PeerJ* **7**, e8169 (2019).

2. Isola, P., Xiao, J., Torralba, A. & Oliva, A. What makes an image memorable? 8 (2011).

3. Isola, P., Xiao, J., Parikh, D., Torralba, A. & Oliva, A. What Makes a Photograph Memorable? *IEEE Trans. Pattern Anal. Mach. Intell.* **36**, 1469–1482 (2014).

4. Khosla, A., Raju, A. S., Torralba, A. & Oliva, A. *Understanding and Predicting Image Memorability at a Large Scale*. http://memorability.csail.mit.edu (2015).

5. Needell, C. D. & Bainbridge, W. A. Embracing New Techniques in Deep Learning for Estimating Image Memorability. (2021).

6. Li, X., Bainbridge, W. A. & Bakkour, A. Item memorability has no influence on value-based decisions. *Sci. Rep. 2022 121* **12**, 1–14 (2022).

7. Võ, M. L.-H., Bylinskii, Z. & Oliva, A. Image Memorability in the Eye of the Beholder: Tracking the Decay of Visual Scene Representations. *bioRxiv* 141044 (2017) doi:10.1101/141044.

8. Zhao, C., Fukuda, K., Park, S. & Woodman, G. F. Even affective changes induced by the global health crisis are insufficient to perturb the hyper-stability of visual long-term memory Significance statement. *Cogn. Res. Princ. Implic.* 7–62 (2022) doi:10.1186/s41235-022-00417-2.

9. Zoom Video Communications Inc. Zoom. Zoom Video Communications Inc (2022).

10. Brainard, D. H. The Psychophysics Toolbox. *Spat. Vis.* **10**, 433–436 (1997).

11. Peirce, J. *et al.* PsychoPy2: Experiments in behavior made easy. *Behav. Res. Methods* **51**, 195–203 (2019).

12. Stark, C. E. L., Noche, J. A., Ebersberger, J. R., Mayer, L. & Stark, S. M. Optimizing the mnemonic similarity task for efficient, widespread use. *Front. Behav. Neurosci.* **17**, (2023).

13. Stark, S. M., Yassa, M. A., Lacy, J. W. & Stark, C. E. L. A task to assess behavioral pattern separation (BPS) in humans: Data from healthy aging and mild cognitive impairment. (2013) doi:10.1016/j.neuropsychologia.2012.12.014.

14. Ezzyat, Y. & Davachi, L. Similarity breeds proximity: pattern similarity within and across contexts is related to later mnemonic judgments of temporal proximity. *Neuron* **81**, 1179–1189 (2014).

15. Morales-Calva, F. & Leal, S. L. Emotional Modulation of Memorability in Mnemonic Discrimination. *Neurobiol. Learn. Mem.* (2024) doi:https://doi.org/10.1016/j.nlm.2024.107904.

16. Rust, N. C. & Jannuzi, B. G. L. Identifying Objects and Remembering Images: Insights From Deep Neural Networks. *Curr. Dir. Psychol. Sci.* **31**, 316–323 (2022).

17. Rust, N. C. & Mehrpour, V. Understanding Image Memorability. *Trends Cogn. Sci.* **24**, 557–568 (2020).

18. Hodes, E., Dement, W. & Zarcone, V. The development and use of the Stanford Sleepiness Scale (SSS). *Psychophysiology* **9**, (1972).

19. Buysse, D. J., Reynolds, C. F., Monk, T. H., Berman, S. R. & Kupfer, D. J. The Pittsburgh Sleep Quality Index: a new instrument for psychiatric practice and research. *Psychiatry Res.* **28**, 193–213 (1989).

20. Beck, A. T., Ward, C. H., Mendelson, M., Mock, J. & Erbaugh, J. An inventory for measuring depression. *Arch. Gen. Psychiatry* **4**, 561–571 (1961).

21. Beck, A. T., Epstein, N., Brown, G. & Steer, R. A. An inventory for measuring clinical anxiety: psychometric properties. *J. Consult. Clin. Psychol.* **56**, 893–897 (1988).

22. Posner, K. *et al.* The Columbia–Suicide Severity Rating Scale: Initial Validity and Internal Consistency Findings From Three Multisite Studies With Adolescents and Adults. *Am. J. Psychiatry* **168**, 1266–1277 (2011).

23. Derryberry, D. & Reed, M. A. Anxiety-related attentional biases and their regulation by attentional control. *J. Abnorm. Psychol.* **111**, 225–236 (2002).

24. Kessler, R. C. *et al.* The World Health Organization Adult ADHD Self-Report Scale (ASRS): a short screening scale for use in the general population. *Psychol. Med.* **35**, 245–256 (2005).

25. Ustun, B. *et al.* The World Health Organization Adult Attention-Deficit/Hyperactivity Disorder Self-Report Screening Scale for DSM-5. *JAMA Psychiatry* **74**, 520–526 (2017).

26. McWhorter, K., Severson, H. & Jones, J. M. *Study and Critical Thinking Skills in College*. (Pearson Education, 2011).

27. Canfield, A. A. & Knight, W. Learning Styles Inventory. (1983).

28. Brickenkamp, R. & Zillmer, E. *The D2 Test of Attention*. (Hogrefe & Huber, Seattle, 1998).

29. Mannion, R., Harikumar, A., Morales-Calva, F. & Leal, S. L. A novel face-name mnemonic discrimination task with naturalistic stimuli. *Neuropsychologia* **189**, 108678 (2023).
